# Combined Piezoelectric and Laser Therapy for Severely Compromised Teeth: Two Case Reports

**DOI:** 10.1155/crid/6024213

**Published:** 2026-07-22

**Authors:** Hyun Nyun Woo, Yong Tak Jeong, Hyun Woo Cho, Jae Mok Lee, Sung-Min Hwang, Young Joon Cho

**Affiliations:** ^1^ Private Practice, New York, New York, USA; ^2^ Mac Dental Clinic, Daegu, Republic of Korea; ^3^ Department of Periodontology, School of Dentistry, Kyungpook National University, Daegu, Republic of Korea, knu.ac.kr; ^4^ Department of Oral and Maxillofacial Surgery, Boston University Henry M. Goldman School of Dental Medicine, Boston, Massachusetts, USA, bu.edu

**Keywords:** case reports, Nd:YAG lasers, periodontal attachment loss, tooth mobility, ultrasonic surgical procedures

## Abstract

**Background:**

Deep periodontal defects (probing depths: 10–15 mm) are traditionally considered hopeless and are frequently recommended for extraction. This report describes two patients demonstrating substantial periodontal tissue healing using a minimally invasive, nonsurgical approach combining piezoelectric instrumentation with Nd:YAG laser therapy, without bone grafts or biologic adjuncts.

**Methods:**

Two systemically healthy, nonsmoking adults with periodontally hopeless teeth underwent subgingival scaling with chlorhexidine irrigation, followed by Nd:YAG laser therapy. Case 1 received delayed laser therapy at 6 months; Case 2 received immediate combined therapy for an acute periodontal abscess. Teeth were stabilized by splinting. Clinical parameters, including probing depth, clinical attachment level (CAL), gingival recession, bleeding on probing, and gingival and plaque indices, were recorded at baseline and follow‐up. Periapical radiographs were qualitatively evaluated for bone density changes.

**Results:**

Case 1 showed a 90.0% reduction in probing depth (10 mm to 1 mm) at 12 months, with a 9‐mm CAL gain (15 mm to 6 mm; 60.0% improvement) and stable recession. Case 2 demonstrated a 93.3% reduction at most sites (15 mm to 1 mm) by 4 months, with a 14‐mm CAL gain (20 mm to 6 mm; 70.0% improvement) and stable recession; however, one site retained a 4‐mm probing depth with a persistent vertical defect. Radiographs revealed increased bone density and re‐established trabecular patterns. No immediate postoperative pain or swelling was reported.

**Conclusion:**

Combined piezoelectric and Nd:YAG laser therapy was associated with substantial clinical and radiographic improvement in periodontally hopeless teeth, suggesting a minimally invasive alternative to extraction in selected cases with severe attachment loss, when bacterial control and patient compliance are favorable.

**One Sentence Summary**

Combined piezoelectric instrumentation and Nd:YAG laser periodontal therapy were associated with marked clinical and radiographic improvement consistent with periodontal healing in hopeless teeth with extreme PDs without conventional bone grafts or biologic materials.

**Key Points**

• Combined piezoelectric instrumentation and Nd:YAG laser therapy may enable preservation of teeth traditionally considered hopeless.
• Significant reductions in probing depth (PD) and clinical attachment gains can be achieved without flap surgery or grafting materials.
• This minimally invasive approach may serve as a conservative alternative to extraction in selected, compliant patients.

**Plain Language Statement**

Severe gum disease often leads to advanced bone loss around teeth, leaving clinicians with no choice but to extract them. Although conventional surgeries can help save some teeth, they are often painful and cause the gums to shrink. This study presents two successful cases where severely damaged teeth, initially deemed “hopeless” and scheduled for extraction, were completely saved without traditional surgery. Instead, the authors combined two modern dental technologies: high‐frequency ultrasonic cleaning devices that gently break down bacterial biofilms and a specialized laser system that selectively vaporizes diseased tissue while leaving healthy areas unharmed. Over a 12‐month follow‐up, both patients experienced dramatic bone healing and gum reattachment, allowing them to chew comfortably without any postoperative pain or swelling. This approach highlights a promising, patient‐friendly alternative to surgery, demonstrating that even teeth on the brink of extraction can be successfully preserved through a minimally invasive combination of ultrasonic and laser energy.

## 1. Introduction

Periodontal disease affects approximately 11.2% of the global population, with severe periodontitis causing substantial tooth loss [1]. The goal of periodontal therapy has evolved from disease control to periodontal regeneration, which aims to restore lost supporting structures, including cementum, periodontal ligament, and alveolar bone [2]. Traditionally, treatment algorithms recommend extraction for teeth with probing depth (PD) exceeding 10–12 mm, as these are considered “hopeless” with poor long‐term prognosis [2, 3]. However, the complications and costs associated with extraction and implant replacement have driven interest in conservative regenerative alternatives.

Minimally invasive modalities, such as piezoelectric ultrasonic instrumentation and laser therapy, have emerged as promising regenerative approaches. Piezoelectric devices enable selective hard tissue removal and improved access to deep pockets, whereas cavitation enhances bacterial reduction [4–10]. Previous studies have reported favorable clinical outcomes using piezoelectric tips in nonsurgical settings [7–9]. In a similar vein, the laser‐assisted new attachment procedure (LANAP), which utilizes a free‐running pulsed 1064‐nm Nd:YAG laser system^1^, holds regulatory clearance from the Food and Drug Administration (FDA) specifically for the regeneration of the periodontium, backed by microscopic proof of de novo attachment apparatus creation [11–14]. This specific comprehensive protocol is structured as a multistep procedure: It begins with initial laser selective debridement to ablate infected pocket epithelium and denature specific periodontopathogens, followed by specialized piezoelectric subgingival scaling for meticulous root surface instrumentation, and concludes with a secondary laser pass designed to thermalize the blood, thereby engineering a stable fibrin clot at the gingival margin [11–13]. Furthermore, contemporary clinical trials have validated that incorporating this specialized laser protocol alongside conventional root surface debridement yields highly favorable outcomes when managing advanced periodontal destruction [12, 13]. Compared with other dental laser wavelengths, the 1064‐nm Nd:YAG laser possesses distinct optical characteristics that offer significant clinical advantages in periodontal therapy. Unlike Er:YAG or CO_2_ lasers, which are highly absorbed by water and hydroxyapatite and thus primarily act on superficial surfaces, the 1064‐nm wavelength exhibits low water absorption. This allows it to penetrate deeply into the soft tissues (up to several millimeters), effectively targeting pathogens harbored within the deep epithelial lining and connective tissue of the periodontal pocket. In addition, its energy is selectively absorbed by dark‐pigmented chromophores, such as hemoglobin and melanin. This selectivity enables the precise destruction of major black‐pigmented periodontopathogens (e.g., *Porphyromonas gingivalis*) and the ablation of diseased pocket epithelium, while strictly safeguarding the underlying healthy connective tissue and alveolar bone. Consequently, this deep‐penetrating, selective mechanism promotes superior pocket debridement and facilitates stable fibrin clot formation, which is essential for subsequent attachment gain.

Despite these advances, existing literature on laser‐ and piezoelectric‐assisted therapy primarily focuses on moderate defects or incorporates adjunctive bone grafts and biologic materials [15]. Evidence regarding the tissue healing of severe periodontal defects (≥ 10 mm) using purely minimally invasive approaches remains limited. Furthermore, previous reports have not adequately explored the synergistic effects of combining piezoelectric cavitation with specific laser protocols in “hopeless” cases without grafting.

Conventional nonsurgical periodontal therapy has limitations in deep intrabony defects due to restricted instrument access and persistent bacterial reservoirs within the pocket lining [9, 11]. Recent advancements emphasize minimally invasive strategies that exploit the biological synergy of ultrasonic cavitation and laser–tissue interactions. Specifically, combining piezoelectric instrumentation with Nd:YAG laser therapy facilitates both mechanical debridement and biological stimulation without the morbidity associated with flap surgery [11–14].

This report presents two cases—utilizing both delayed and immediate approaches—where periodontally hopeless teeth were successfully preserved. The protocol integrated mechanical stabilization through splinting and adjunctive systemic medication, while primarily focusing on the synergistic regenerative effects of electro medical systems (EMS) piezoelectric instrumentation with chlorhexidine irrigation and Nd:YAG laser therapy. By achieving exceptional management in 10–15‐mm defects without bone grafts, these cases broaden current understanding of nonsurgical therapy and provide a potential clinical framework for preserving severely compromised teeth.

## 2. Materials and Methods

### 2.1. Design and Ethical Considerations

This retrospective case report describes two patients with periodontally hopeless teeth (PDs: ≥ 10 mm) treated using combined piezoelectric instrumentation and Nd:YAG laser periodontal therapy. Both patients provided written informed consent for treatment and publication of anonymized clinical data, including radiographs. Institutional Review Board (IRB) approval was not required for this study according to the institutional guidelines of the Western Institutional Review Board‐Copernicus Group (WCG IRB) and U.S. Federal Regulations (45 CFR 46). This report qualifies for exemption as a retrospective analysis of two clinical cases utilizing fully anonymized, de‐identified data with obtained written informed consent and no risk of disclosing patient identity. Treatment followed standard clinical practice guidelines and adhered to the Declaration of Helsinki. Both patients provided written informed consent prior to the procedures for the performance of all clinical therapies, the utilization of their de‐identified clinical records, and the publication of this case report along with the accompanying radiographs. The manuscript was prepared in accordance with the CARE (Case Report) guidelines [16].

### 2.2. Clinical Examination, Radiographic Evaluation, and Documentation

Clinical parameters were recorded at baseline and each follow‐up visit. Tooth numbering follows the two‐digit notation system of the FDI World Dental Federation (Fédération Dentaire Internationale). PD was measured using a standard periodontal probe at six sites per tooth (mesiobuccal, buccal, distobuccal, mesiolingual, lingual, and distolingual). Gingival recession was defined as the distance from the cementoenamel junction (CEJ) to the gingival margin. Clinical attachment level (CAL) was calculated as the sum of PD and recession. Bleeding on probing (BOP), gingival index [17] (0–3 scale), and plaque index [18] (0–3 scale) were assessed using established criteria. Tooth mobility was graded 0–3 according to Miller′s classification [19] before splinting. Following splint placement, tooth mobility could not be independently assessed and was recorded as “not available” (N/A) at all subsequent visits. Periapical radiographs were obtained at baseline and final follow‐up using a standardized paralleling technique with a film holder to ensure reproducibility. Due to the retrospective design and limitations of two‐dimensional imaging, radiographic bone changes were assessed qualitatively. Bone density and trabecular organization were evaluated visually and compared between baseline and follow‐up radiographs. The presence of periapical radiolucency, vertical bone defects, and overall changes in bone density were documented. Precise quantitative and three‐dimensional assessment of the defects was not possible.

### 2.3. Postoperative Management

Both patients received standardized postoperative medications, including amoxicillin (875 mg every 12 h for 8 days), ibuprofen (600 mg every 8 h as needed for 8 days), and chlorhexidine gluconate 0.12% rinse (15 mL for 30 s twice daily for 2 weeks). Instructions included a soft diet, avoidance of straws, and restriction of vigorous rinsing for 2 weeks. A 1‐day postoperative telephone follow‐up assessed pain, swelling, analgesic use, and compliance with postoperative instructions. Clinical re‐evaluation was performed at 2 weeks. Periodontal maintenance was scheduled at 3‐month intervals following completion of regenerative therapy. Intervention adherence was assessed through follow‐up communication, attendance at re‐evaluation visits, and plaque control at follow‐up. Tolerability was assessed by postoperative telephone follow‐up and subsequent clinical re‐evaluation.

### 2.4. Case 1: Delayed Piezoelectric and Nd:YAG Laser Therapy for a Hopeless Mandibular Anterior Tooth

A 60‐year‐old woman was referred by a general dentist for periodontal consultation in Westchester County, New York, in May 2025, with a chief complaint of a loose lower anterior tooth. A periodontally hopeless tooth 41 exhibited 10‐mm PD on all buccal sites, Grade 3 mobility, severe radiographic bone loss with reduced density and disrupted trabecular pattern, CAL of 15 mm, gingival recession of 5 mm, gingival index of 3, and plaque index of 3 (Figure 1A). The clinical periodontal diagnosis was severe localized periodontal destruction involving tooth 41 with a hopeless periodontal prognosis based on extreme PD, severe CAL loss, mobility, and radiographic bone loss. Pulp vitality testing revealed a positive response to cold stimulation. There was no history of smoking, uncontrolled systemic disease, or other relevant medical conditions affecting periodontal treatment. Relevant family/genetic history was not contributory based on the available records, and no psychosocial barriers to treatment adherence were identified. The patient was informed of the hopeless prognosis and extraction recommendation according to conventional guidelines but expressed a strong preference for tooth preservation before considering extraction. No other relevant prior periodontal intervention for the involved tooth was documented before the present treatment.

**Figure 1:**
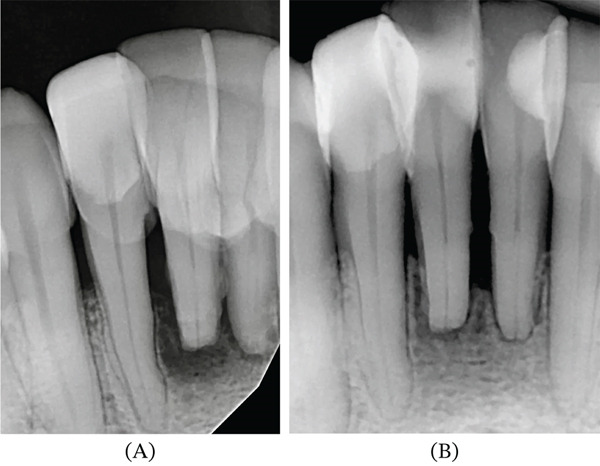
Case 1: periapical radiographs. (A) Baseline radiograph showing severe bone loss with reduced bone density and disrupted trabecular pattern. (B) Twelve‐month radiograph demonstrating increased bone density and re‐established trabecular architecture, suggestive of radiographic bone fill and remodeling.

A direct resin bonding splint extending from tooth 33 to tooth 43 was placed to reduce mobility. No occlusal adjustment was performed. Subgingival scaling was performed using a piezoelectric ultrasonic scaler unit^2^ at power Level 5 with continuous irrigation using chlorhexidine gluconate 0.12% diluted 1:3 with sterile distilled water. Hand curettes were not used. Specialized periodontal ultrasonic tips (a fine‐bladed tip, a diamond‐coated furcation tip, and a universal scaling tip)^3^ were systematically applied to all root surfaces, with 3–5 min of treatment per tooth. Local anesthesia (2% lidocaine with 1:100,000 epinephrine) was administered. Standard postoperative medications were prescribed, and home care instructions reinforced a soft diet, avoidance of straws, and rinsing precautions for 2 weeks.

The patient was unavailable for the scheduled 3‐month periodontal maintenance visit due to travel. At 6 months, re‐evaluation led to the addition of a free‐running pulsed 1064‐nm Nd:YAG laser system^4^. Local anesthesia (2% lidocaine with 1:100,000 epinephrine) was administered. The first laser phase was delivered at 3.60 W, 20 Hz, 180 mJ, 100 *μ*s pulse duration, using a 360‐*μ*m fiber. Two systematic passes per pocket wall were performed from the gingival margin to the apical extent, at 3–4 s per site, ensuring complete fiber exit with each pass. The same piezoelectric scaling protocol was then repeated. Following debridement, mechanical stimulation of the alveolar bone and periodontal ligament was performed using the ultrasonic tip to induce controlled bleeding from the bone and periodontal ligament space. A second Nd:YAG laser phase was applied at 3.6 W, 20 Hz, 180 mJ, 550 *μ*s pulse duration, using a 360‐*μ*m fiber. Two systematic passes per pocket wall were performed from the apical extent to the gingival margin, at 3–4 s per site, with complete fiber exit. Total energy (J) was recorded ^5^after both laser phases. No occlusal adjustment was performed to preserve fibrin clot stability and the regenerative environment created. Mechanical stability was maintained with the existing splint, which controlled tooth mobility and occlusal forces. The standard postoperative medication protocol was prescribed. Periodontal maintenance was scheduled and performed at 3‐month intervals.

### 2.5. Case 2: Immediate Piezoelectric and Nd:YAG Laser Therapy for a Hopeless Mandibular Lower Anterior Tooth With Acute Periodontal Abscess

A 56‐year‐old man presented with a chief complaint of a mobile lower anterior tooth with an abscess referred by a general dentist in Western County, New York in November 2025. A periodontally hopeless tooth 31 exhibited 15‐mm PD on all buccal sites, Grade 3 mobility, and an active periodontal abscess on the buccal surface. Radiographic evaluation showed severe bone loss with periapical radiolucency and significantly reduced bone density. CAL was 20 mm, gingival recession 5 mm, gingival index 3, and plaque index 2 (Figure 2A). The clinical diagnosis was severe localized periodontal destruction with acute periodontal abscess involving tooth 31 and a hopeless periodontal prognosis. Given the history of unknown endodontic vitality and prior endodontic treatment and the persistent lesion, possible residual endodontic involvement was considered; however, the case was managed primarily as a severe periodontal lesion with acute abscess. The patient was a nonsmoker and had no reported uncontrolled systemic disease relevant to periodontal healing. Relevant family/genetic history was not contributory based on the available records, and no psychosocial factors limiting treatment participation were identified. Endodontic treatment and wire splinting had been completed previously; however, the abscess persisted. The patient was informed of the poor prognosis and extraction recommendation but strongly preferred tooth preservation and requested conservative treatment.

**Figure 2:**
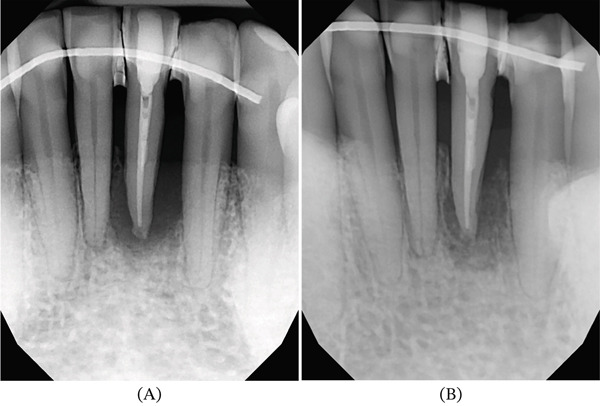
Case 2: periapical radiographs and qualitative bone density analysis. (A) Baseline radiograph showing severe bone loss with periapical radiolucency, active abscess, and reduced bone density. (B) Four‐month radiograph demonstrating substantially increased bone density at other sites, re‐established trabecular architecture, and complete resolution of periapical radiolucency. A persistent vertical bone defect is visible at the buccodistal aspect, corresponding to a 4‐mm probing depth.

After written and verbal consent, local anesthesia was administered (2% lidocaine with 1:100,000 epinephrine). The existing splint maintained stable occlusion by redistributing occlusal forces and providing stabilization. The same protocol as in Case 1 was followed, using the Nd:YAG laser system^6^ and subgingival scaling with piezoelectric ultrasonic device^7^. The standard postoperative medication protocol was prescribed.

### 2.6. Outcome Measures

Primary outcome measures included changes in PD and CAL from baseline to final follow‐up. Secondary outcomes comprised gingival recession, BOP, gingival inflammation (gingival index), plaque accumulation (plaque index), tooth mobility (when assessable prior to splinting), and qualitative radiographic bone changes, including density and trabecular organization. Patient‐reported outcomes, including postoperative pain, swelling, analgesic use, and functional comfort, were recorded through telephone follow‐up and clinical examination.

### 2.7. Data Presentation and Analysis

Clinical parameters are presented as absolute values (mm) at each time point, with changes from baseline expressed as absolute differences (*Δ* mm) and percentages (*Δ*%). For PD and CAL, negative values indicate reduction, whereas positive values indicate gain. Given the retrospective case report design involving two patients, no statistical analysis was performed. Results are presented descriptively, with individual case timelines, comprehensive data tables, and graphical representations of clinical parameter changes over time. Radiographic outcomes are described qualitatively through comparative visual assessment between baseline and follow‐up images.

### 2.8. Follow‐Up Protocol

Case 1 was followed for 12 months, with assessments at baseline, 6 (laser intervention), 9, and 12 months. Case 2 was followed for 4 months, with assessments at baseline (immediate laser intervention), 2 weeks (abscess resolution evaluation), and 4 months. These differing follow‐up durations reflect the retrospective design and timing of presentation. Three‐month periodontal maintenance visits were performed following completion of regenerative therapy to maintain plaque control and monitor treatment stability.

## 3. Results

Healing was uneventful, with minimal postoperative morbidity reported by both patients. In Case 1, at 6 months, PD was reduced to 5 mm (50.0% reduction from baseline), with stable recession of 5 mm, minimal inflammation, and a CAL of 10 mm, reflecting a 5‐mm gain (33.3% improvement). Tooth mobility could not be independently assessed due to the intact splint. Following Nd:YAG laser therapy at 6 months, the 12‐month evaluation showed further improvement, with PD reduced to 1 mm (90.0% reduction from baseline), stable gingival recession of 5 mm, and CAL of 6 mm, corresponding to a 9‐mm gain from baseline (15 mm; 60.0% improvement). Gingival index and plaque index were both 0, and BOP was absent. The 9‐mm CAL gain, with stable recession, indicated substantial restoration of periodontal support while preserving gingival margin position (Table 1, Figure 3). Radiographic evaluation demonstrated increased bone density and re‐establishment of organized trabecular architecture compared with baseline, suggesting radiographic bone fill and remodeling compatible with periodontal healing (Figure 1). The patient reported no discomfort throughout follow‐up and maintained excellent oral hygiene. The splint remained intact during the follow‐up period.

**Table 1:** Case 1: clinical parameters at baseline and follow‐up intervals.

Parameter	Baseline	12‐month	*Δ* value	*Δ*%
PD (mm)	10	1	9	90.0
Recession (mm)	5	5	0	0
CAL (mm)	15	6	9	60.0
Radiographic bone density	Severe bone loss	Radiographic improvement	—	—
GI (0–3)	3	0	3	100
PI (0–3)	3	0	3	100
BOP	Present	Absent	—	—
Mobility	3	N/A	—	—

*Note:* Radiographic bone density assessed qualitatively.

Abbreviations: *Δ*, delta (change from baseline); BOP, bleeding on probing; CAL, clinical attachment level; GI, gingival index; N/A, not available; PD, probing depth at affected buccal sites; PI, plaque index.

**Figure 3:**
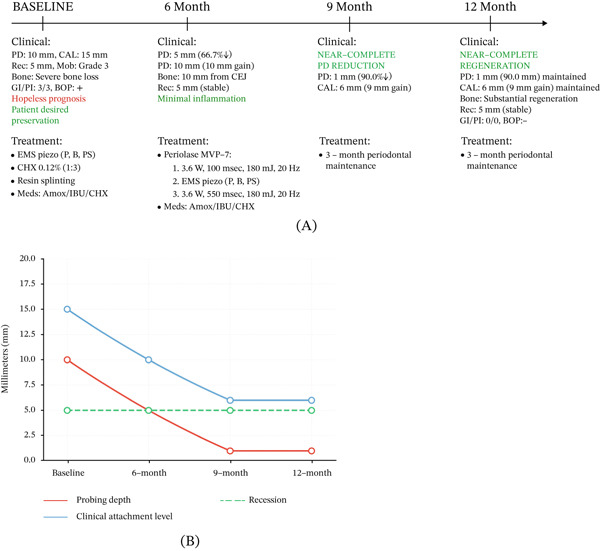
Case 1: treatment timeline and clinical parameters. (A) Flowchart illustrating the treatment protocol from baseline through 12‐month follow‐up with delayed laser therapy at 6 months. (B) Line graph displaying probing depth (PD), clinical attachment level (CAL), and recession over time.

In Case 2, a 1‐day postoperative follow‐up revealed no pain, swelling, or need for ibuprofen. At 2 weeks, clinical signs of acute inflammation, including swelling and suppuration, had resolved completely; the patient reported no pain. PD was 10 mm (33.3% reduction from baseline), with stable gingival recession of 5 mm and CAL of 15 mm, reflecting a 5‐mm gain (25.0% improvement) and minimal inflammation. Tooth mobility could not be assessed due to pre‐existing wire splinting. At 4 months, PD was reduced to 1 mm at most sites (93.3% reduction), with a residual 4‐mm depth at the buccodistal aspect of tooth 31, averaging 2.5 mm overall (83.3% reduction). Gingival recession remained stable at 5 mm. CAL was 6 mm at most sites, indicating a 14‐mm gain from baseline (20 mm; 70.0% improvement) and 9 mm at the buccodistal site, indicating an 11‐mm gain (55.0% improvement), averaging a 7.5‐mm gain (62.5% improvement). Gingival index and plaque index were both 0, and BOP was absent at the final follow‐up. Clinical attachment gains of up to 14 mm at most sites, with stable gingival recession, indicated marked clinical improvement without additional gingival margin loss (Table 2, Figure 4). Radiographic examination revealed increased radiopacity at previous defect sites, with re‐establishment of the normal trabecular architecture and resolution of the periapical radiolucency. A localized vertical bone defect persisted at the distobuccal aspect, consistent with the residual 4 mm PD; however, no recurrence of infection was observed. The pre‐existing wire splint provided continuous stabilization throughout follow‐up (Figure 2).

**Table 2:** Case 2: clinical parameters at baseline and follow‐up intervals.

Parameter	Baseline	4‐month (other sites)	4‐month (BD site)	Mean *Δ*	Mean *Δ*%
PD (mm)	15	1	4	12.5	83.3
Recession (mm)	5	5	5	0	0
CAL (mm)	20	6	9	7.5	62.5
Radiographic bone density	Severe bone loss	Radiographic improvement	Radiographic improvement	—	—
GI (0–3)	3	0	0	3	100
PI (0–3)	2	0	0	2	100
BOP	Present	Absent	Absent	—	—
Mobility	3∗	N/A	N/A	—	—
Abscess	Present	Absent	Absent	—	—

*Note:* Asterisk “∗” denotes mobility Grade 3 assumed prior to splinting. Radiographic bone density was assessed qualitatively.

Abbreviations: *Δ*, delta (change from baseline); BD, buccodistal; BOP, bleeding on probing; CAL, clinical attachment level; GI, gingival index; N/A, not available; PD, probing depth at affected buccal sites; PI, plaque index.

**Figure 4:**
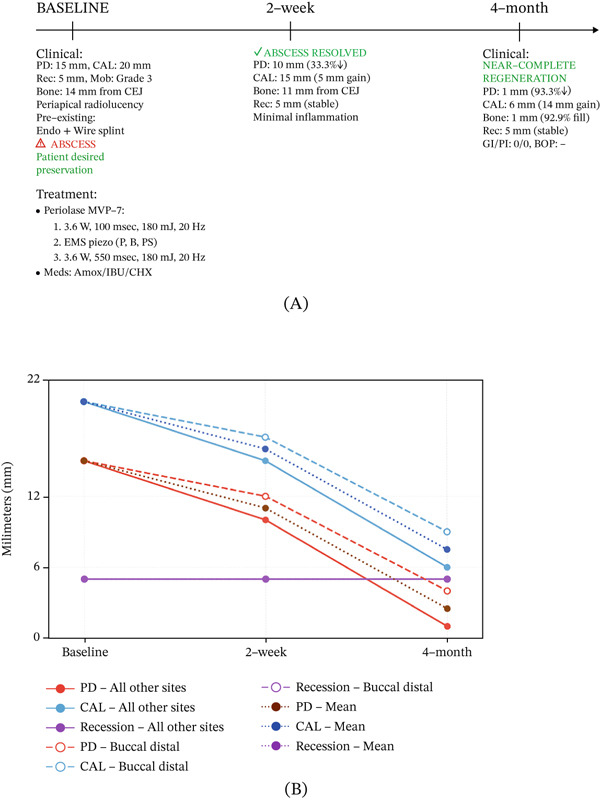
Case 2: treatment timeline and clinical parameters with site comparison. (A) Flowchart illustrating the treatment protocol, including immediate laser therapy and the timeline of abscess resolution. (B) Combined line graph displaying probing depth, clinical attachment level, and gingival recession at the other sites and the buccodistal site of tooth 31, together with the corresponding mean values.

## 4. Discussion

These cases showed marked clinical and radiographic improvement after minimally invasive nonsurgical therapy combining piezoelectric instrumentation and Nd:YAG laser treatment. PD reductions of 73.3%–93.3% and CAL gains of 9–14 mm were observed at most sites, with stable gingival recession and improved radiographic bone density. The observed CAL gains, with stable gingival recession (5 mm), suggest restoration of connective tissue attachment to the root surface rather than apical migration of the junctional epithelium. These outcomes considerably exceed conventional expectations, which typically recommend extraction for PDs exceeding 10–12 mm [2, 3].

The magnitude of CAL gain (9–14 mm) with stable recession provides quantitative evidence suggestive of periodontal repair and regeneration. This clinical outcome likely reflects new connective tissue attachment with underlying bone support rather than reduced PD due to long junctional epithelium formation alone. Radiographic evaluation demonstrated substantial improvements in bone density and trabecular organization in both cases. Although precise linear measurements from periapical radiographs are limited by angulation variability and two‐dimensional imaging constraints, the increased radiodensity and re‐established trabecular architecture strongly indicate significant clinical and radiographic improvement that is highly suggestive of hard tissue remodeling. In Case 2, complete resolution of the periapical radiolucency further supports successful bone healing.

The exclusive use of piezoelectric instrumentation^8^ at power Level 5, without hand curettes, represents an advancement in minimally invasive periodontal therapy. Operating via linear oscillations at a frequency range of 25,000–30,000 Hz, piezoelectric units provide clinicians with superior tactile feedback, expedite the instrumentation process, and alleviate patient discomfort while inducing critical cavitation dynamics [4, 5]. This cavitation process precipitates the formation of minute bubbles that subsequently implode adjacent to the subgingival microflora, thereby disintegrating the bacterial cellular envelopes through the dual actions of acoustic microstreaming and localized oxidative stress [11]. The use of chlorhexidine gluconate 0.12%, diluted 1:3 with distilled water as an irrigant, provides additional antimicrobial activity against periodontal pathogens through bacterial cell membrane disruption and substantivity [20].

The technical framework of an Nd:YAG laser system^9^ utilizes a free‐running pulsed Nd:YAG laser source operating at a 1064‐nm wavelength tailored for intraoral soft‐tissue therapy. Owing to its high affinity for dark‐colored chromophores such as melanin and hemoglobin—coupled with minimal attenuation in hydroxyapatite and water—this specific wavelength allows for the targeted ablation of inflamed sulcular linings and dark‐pigmented periodontopathogens while safeguarding adjacent healthy anatomical complexes [6]. FDA clearance of LANAP, supported by histological evidence of new cementum, periodontal ligament, and bone formation, provides regulatory support for its regenerative potential [11].

Potential biological mechanisms associated with Nd:YAG laser therapy may extend beyond disinfection. As illustrated in Figure 5, the process involves synergistic phases. First, piezoelectric cavitation disrupts biofilm and enhances root surface decontamination. Moreover, mechanical stimulation of the intrabony defect and periodontal ligament space induces controlled bleeding, mobilizing regenerative cellular and molecular components—including mesenchymal stem cells and endogenous growth factors—into the defect site. Subsequently, the Nd:YAG laser application focuses on the precise elimination of the ulcerated pocket epithelium while protecting the underlying viable connective tissue. This energy delivery leads to the establishment of an intact, secure fibrin coagulum at the crestal margin, which serves as a physiological seal preventing the apical migration of junctional epithelium, mimicking the core principle of guided tissue regeneration [11–14]. By compartmentalizing the subgingival space, this stable clot successfully sequesters localized signaling molecules and pluripotential stem cells, fostering an optimal niche for true periodontal tissue synthesis. Third, photobiomodulation at a wavelength of 1064 nm promotes proliferation of periodontal ligament cells, osteoblasts, and undifferentiated progenitor cells, enhances metabolic activity, and stimulates release of vascular endothelial growth factor (VEGF), facilitating angiogenesis [15, 21, 22]. This regenerative effect is supported by human histological evidence demonstrating formation of a new attachment apparatus following this protocol [11, 14].

**Figure 5:**
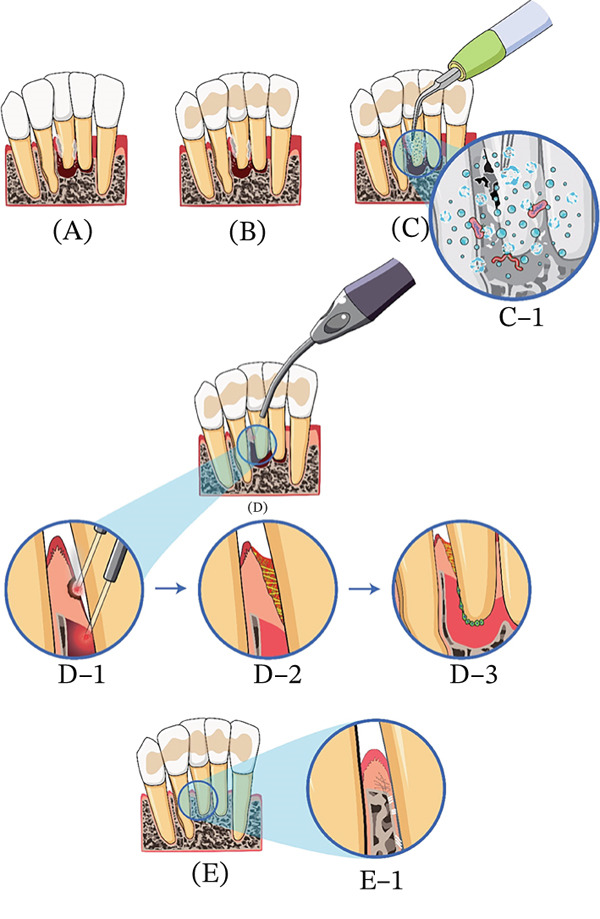
Schematic Illustration of the Minimally Invasive Regenerative Protocol and Its Biological Mechanisms. (A) Baseline defect; (B) Splinting; (C) Piezoelectric debridement with (C‐1) cavitation effect; (D) Nd:YAG laser therapy showing (D‐1) selective debridement, (D‐2) biological seal formation, and (D‐3) photobiomodulation; and (E) Possible repair/regeneration response with (E‐1) an enlarged schematic view of the proposed healing response within the periodontal defect.

The timing of laser therapy differed between cases: Case 1 received delayed treatment at 6 months due to scheduling constraints, whereas Case 2 received immediate combined therapy. Both approaches yielded favorable outcomes; however, the rapid early response in Case 2, with PD reduction from 15 to 10 mm within 2 weeks, suggests potential advantages of immediate laser application in acute presentations. The acute periodontal abscess in Case 2 resolved completely within 2 weeks following combined piezoelectric‐chlorhexidine–laser therapy and systemic antibiotics, without surgical drainage, indicating effective nonsurgical management of severe acute periodontal infections [23]. Endodontic status was monitored collaboratively with the referring dentist throughout follow‐up, and no pulpal complications occurred.

The achievement of substantial regeneration without grafting materials challenges assumptions regarding the necessity of bone grafts, barrier membranes, or biologic materials in managing severe defects. Comprehensive bacterial reduction, preservation of soft tissue architecture through a minimally invasive approach, laser‐induced biomodulation, mechanical stabilization via splinting, and strong patient compliance likely contributed to the favorable regenerative response [24]. Although the official LANAP protocol strictly dictates subtractive occlusal adjustment as an essential step to protect the healing environment, a modified mechanical stabilization strategy was intentionally adopted in the present cases. Given that the targeted mandibular incisors exhibited extreme bone loss with Grade 3 mobility, simply reducing the occlusal height would be insufficient to counteract harmful lateral forces during mastication. Therefore, rigid splinting was utilized as an alternative to conventional occlusal adjustment. This approach effectively redistributed traumatic occlusal forces across the entire anterior segment, successfully achieving the ultimate therapeutic goal of protecting the delicate intrapocket fibrin clot and securing an undisturbed regenerative environment. While the stability of the 5‐mm gingival recession alone cannot definitively rule out the histological formation of a long junctional epithelium or simple reattachment, the significant CAL gains accompanied by corresponding radiographic enhancements in bone density strongly suggest a favorable periodontal healing response conducive to new attachment.

The persistent 4‐mm PD, CAL of 9 mm, and radiographically evident vertical one‐wall bone defect at the buccodistal aspect of tooth 31 in Case 2 indicate that complete clinical resolution may not be uniformly achieved. This site‐specific incomplete response may reflect anatomical complexity, variable bacterial colonization, limited instrumentation access, or biological variability. Nevertheless, this residual defect represents a 73.3% reduction in PD and a 55.0% improvement in CAL from baseline, outcomes still considered successful in conventional periodontal therapy.

As patient perspective, both patients expressed high satisfaction with the treatment outcomes and the minimally invasive nature of the procedures. Both patients emphasized that the treatment experience and outcomes far exceeded their expectations given the initial hopeless prognosis. The unremarkable healing phase observed in both individuals, distinguished by an absence of swelling, significant pain, or reliance on oral analgesics, represents a striking departure from typical surgical interventions. This comfortable postoperative recovery is highly attributable to the conservative modality that circumvents periosteal flap elevation and maintains marginal tissue anatomy, paired with laser‐driven photobiomodulation that suppresses the cascade of proinflammatory cytokines within the treated site [25].

Limitations include the small sample size, limiting generalizability; absence of a control group, preventing identification of critical treatment components; differing follow‐up durations, limiting direct comparison; lack of histological confirmation of regeneration; inability to obtain precise quantitative bone measurements from two‐dimensional periapical radiographs; single‐operator design, raising reproducibility concerns; and potential patient selection bias involving highly motivated, compliant patients. Future studies should include larger cohorts, prospective randomized designs, extended follow‐up, histological validation, three‐dimensional cone beam computed tomography for accurate bone assessment, and systematic evaluation of prognostic factors. Emerging scaffold‐based approaches using bioactive materials and hydrogels may further enhance regenerative outcomes when combined with minimally invasive techniques [26].

Recent long‐term data demonstrate favorable tooth retention rates, averaging 0.11 teeth lost per patient per year over a mean follow‐up of 6.7 years after full‐mouth LANAP therapy [27]. These cases provide preliminary evidence that advanced nonsurgical approaches combining piezoelectric instrumentation with antimicrobial irrigation and Nd:YAG laser therapy may expand treatment options for patients at risk of tooth loss. In carefully selected, motivated patients with localized severe disease, a trial of nonsurgical regenerative therapy may be considered before assigning a hopeless diagnosis and proceeding to extraction.

Lastly, due to the retrospective nature of this case report, standardized clinical intraoral photographs capturing the chronological healing stages were N/A. Although this lack of visual documentation is a limitation, the precise treatment parameters, comprehensive qualitative radiographic follow‐ups showing bone density changes, and detailed schematic illustrations provided herein sufficiently delineate the clinical procedure for reproducibility.

## 5. Conclusions

The combination of piezoelectric instrumentation and Nd:YAG laser therapy offers a potential, minimally invasive nonsurgical approach for preserving periodontally hopeless teeth with 10–15 mm PDs. This conservative protocol achieved significant clinical attachment gains and radiographic bone density improvements without the need for flap elevation or bone grafting. While limited to two retrospective cases, these outcomes suggest that this advanced nonsurgical modality can serve as a viable alternative to extraction in carefully selected clinical scenarios.

## Author Contributions

Hyun Nyun Woo: conceptualization, methodology, writing—original draft preparation. Yong Tak Jeong: investigation, data curation. Hyun Woo Cho: software, visualization, investigation. Jae Mok Lee: supervision, validation. Sung‐Min Hwang: supervision, project administration. Young Joon Cho: writing—reviewing and editing, validation. Sung‐Min Hwang and Young Joon Cho contributed equally to this work as coprincipal investigators.

## Funding

No funding was received for this manuscript.

## Disclosure

All authors have read and approved the final version of the manuscript. Dr. Young Joon Cho had full access to all of the data in this study and takes complete responsibility for the integrity of the data and the accuracy of the data analysis.

## Ethics Statement

Institutional Review Board (IRB) approval was not required for this study according to the institutional guidelines.

## Conflicts of Interest

The authors declare no conflicts of interest.

## Endnotes

^1^PerioLase MVP‐7; Millennium Dental Technologies, Inc., Cerritos, California, USA.

^2^Piezon Master 700; EMS, Nyon, Switzerland.

^3^P, B, and PS tips; EMS, Nyon, Switzerland.

^4^PerioLase MVP‐7; Millennium Dental Technologies, Inc., Cerritos, California, USA.

^5^PerioLase MVP‐7; Millennium Dental Technologies, Inc., Cerritos, California, USA.

^6^PerioLase MVP‐7; Millennium Dental Technologies, Inc., Cerritos, California, USA.

^7^Piezon Master 700; EMS, Nyon, Switzerland.

^8^Piezon Master 700; EMS, Nyon, Switzerland.

^9^PerioLase MVP‐7; Millennium Dental Technologies, Inc., Cerritos, California, USA.

## Data Availability

Data generated by the examination and treatment of the presented cases are included in this article. Additional data that support the findings of this report are available from the corresponding author upon reasonable request. The authors confirm that the data supporting the findings of this study are available within the article and/or its supporting materials.
